# Potential Criteria for Frameworks to Support the Evaluation of Innovative Medicines in Upper Middle-Income Countries—A Systematic Literature Review on Value Frameworks and Multi-Criteria Decision Analyses

**DOI:** 10.3389/fphar.2020.01203

**Published:** 2020-08-14

**Authors:** Ivett Jakab, Bertalan Németh, Baher Elezbawy, Melis Almula Karadayı, Hakan Tozan, Sabahattin Aydın, Jie Shen, Zoltán Kaló

**Affiliations:** ^1^ Syreon Research Institute, Budapest, Hungary; ^2^ Syreon Middle East, Alexandria, Egypt; ^3^ İstanbul Medipol University, İstanbul, Turkey; ^4^ Novartis International AG, Basel, Switzerland; ^5^ Center for Health Technology Assessment, Semmelweis University, Budapest, Hungary

**Keywords:** health technology assessment, pharmaceutical, reimbursement, developing country, multiple criteria

## Abstract

**Background:**

Multicriteria Decision Analysis (MCDA), a formal decision support framework, has been growing in popularity recently in the field of health care. MCDA can support pricing and reimbursement decisions on the macro level, which is of great importance especially in countries with more limited resources.

**Objectives:**

The aim of this systematic review was to facilitate the development of future MCDA frameworks, by proposing a set of criteria focusing on the purchasing decisions of single-source innovative pharmaceuticals in upper middle-income countries.

**Methods:**

A systematic literature review was conducted on the decision criteria included in value frameworks (VFs) or MCDA tools. Scopus, Medline, databases of universities, websites of Health Technology Assessment Agencies, and other relevant organizations were included in the search. Double title-abstract screening and double full-text review were conducted, and all extracted data were double-checked. A team of researchers performed the merging and selection process of the extracted criteria.

**Results:**

A total of 1,878 articles entered the title and abstract screening. From these, 341 were eligible to the full-text review, and 36 were included in the final data extraction phase. From these articles 394 criteria were extracted in total. After deduplication and clustering, 26 different criteria were identified. After the merging and selection process, a set of 16 general criteria was proposed.

**Conclusion:**

Based on the results of the systematic literature review, a pool of 16 criteria was selected. This can serve as a starting point for constructing MCDA frameworks in upper middle-income countries after careful adaptation to the local context.

## Introduction

### The Need for Complex Value Assessment in HTA

Resource allocation in health care is a complex process, economic constraints in all countries necessitate a rational priority setting and a transparent decision-making framework (Baltussen and Niessen, 2006). This need called Health Technology Assessment (HTA) into being, a multidisciplinary field of policy analysis using explicit analytical frameworks, with the main purpose to inform technology-related policymaking in health care (Facey et al., 2006). Essentially, HTA aims to provide the best available information regarding various health technologies in a variety of settings (Velasco-Garrido and Busse, 2005; Grutters et al., 2011).

Another key aspect of health care decision making, in line with the multidisciplinary nature of HTA, is that it is not based on a single criterion, but on a set of different criteria, like efficacy or effectiveness, safety, cost-effectiveness, equity or fairness, etc. (Guindo et al., 2012) that are often in conflict with one another (Keeney and Raiffa, 1993). Lack of awareness of the value elements considered across the decision-making continuum as well as lack of transparency in decision making can potentially create tension amongst stakeholders (Tanios et al., 2013). As a result, more and more organizations are proposing and implementing a “value assessment framework” (or “value framework” (VF) for short) for aiding health care decisions with a clear list of criteria that has to be taken into account (Neumann et al., 2018). It has also been a generally accepted principle that health care decision‐making should rely on strong scientific evidence (Rudmik and Drummond, 2013), which called the need for a scientifically justified method to evaluate more than one aspect of the decision-making problem at once. When criteria of a value framework are weighted as opposed to each other and given a specific measurement method (so-called scoring function), of which a summary of scores can be calculated, the framework is called a Multi-Criteria Decision Analysis (MCDA) or a Multiple-Criteria Decision Making (MCDM) tool.

### What Is MCDA?

MCDA is the collective term for those formal decision support approaches, that take multiple criteria into account in an explicit way (Belton and Stewart, 2002). MCDA has already been used in various fields of science, and it has been gaining popularity recently in the field of health care decision making as well (Marsh et al., 2014; Antioch et al., 2017). Key elements of the MCDA structure are: 1) clearly defined and well-structured criteria to assess the alternatives that are being evaluated in the particular decision setting, 2) weights to express the differences in relative importance of various criteria, and 3) the aforementioned scoring functions for converting performance measurements of the analyzed alternatives into scores that can be aggregated to inform the decision-makers on the overall performance of the alternatives (Thokala et al., 2016).

### Potential of MCDA Use in Health Care

MCDA can be used on all levels of decision making in health care. At micro-level decision making when choosing the treatment to be administered to a certain patient, MCDA can be especially helpful for involving the patient’s voice into his/her own treatment decision (Marsh et al., 2017) through shared decision making [e.g. American Society of Clinical Oncology (ASCO) framework (Cherny et al., 2018)]. The mezzo, clinical guideline or hospital-level decision making can also be guided by MCDAs, for example during the development of the hospital formulary list or a new clinical guideline. Last but not least MCDA can be of great help at macro level in structuring risk-benefit assessment to support market authorization decisions (Angelis and Phillips, 2020) or informing reimbursement decisions for different health technologies, health care programs or health priorities (e.g. EVIDEM [Evidence and Value: Impact on Decision Making) framework (Goetghebeur et al., 2008); The Advance Value Framework (Angelis and Kanavos, 2017)].

In general, an MCDA tool can have two potential roles in reimbursement decisions. If an explicit threshold of MCDA final scores is used as a hard decision rule, MCDAs can be used as independent decision-making tools. For example, if the total attainable score of the MCDA is 100 points, a threshold at 60 points can be set above which all assessed health technologies can receive reimbursed status. The other general approach is to use the MCDA framework in a supportive role, without being the single instrument that guides the final decision. This way, MCDA frameworks can be used as part of deliberate processes (Baltussen et al., 2017), or as a way of conducting so-called augmented cost-effectiveness analyses (Garrison et al., 2018).

The International Society of Pharmacoeconomics and Outcomes Research (ISPOR) had a task force dedicated to the topic of MCDA and published two comprehensive reports (Marsh et al., 2016; Thokala et al., 2016) guiding the development of such tools. Wide MCDA-related research has been conducted both regarding the theoretical side, like reviewing the various approaches adopted in health care MCDAs (Marsh et al., 2014), and the methodological side, for example on the selection of the optimal criteria weighting methods (Németh et al., 2019). Some examples have also been published on the practical issues of using MCDA in real-life decision settings, from applying the EVIDEM framework in Lombardy, Italy (Radaelli et al., 2014), through assessing the value of hospital technologies in Hungary (Endrei et al., 2014) or improving the current procurement framework for off-patent pharmaceuticals in Indonesia (Inotai et al., 2018a). Above all, the usefulness of incorporating MCDA in HTA to support transparent and systematic appraisal of health care interventions has been demonstrated in a proof-of-concept study (Goetghebeur et al., 2012). Also, it has been shown that MCDA integrated into HTA allows a consistent approach for appraising health care interventions, by promoting systematic consideration of all decision criteria and the underlying evidence (Tony et al., 2011).

### HTA in Upper Middle-Income Countries

The level of HTA used in reimbursement decisions is uneven throughout countries, often being less developed in upper-middle-income countries compared to high-income ones. Nonetheless, countries with limited resources can spend a lower amount of monetary resources on health care compared to high-income countries, making their allocative decisions more influential for the whole system (Kaló et al., 2016). This situation is often troubled with a worse general health status of the population (Boncz et al., 2014), and insufficient capacity of HTA expertise (Kaló et al., 2013), therefore the need for and the challenges of implementing HTA for health care technologies are even greater in these countries (Sorenson et al., 2009; Inotai et al., 2012). Application of an MCDA tool adapted to local circumstances can be a good option for overcoming these challenges for upper-middle-income countries, especially for innovative, usually expensive pharmaceuticals.

In this study, we aimed to facilitate the development of such transparent decision-making processes. The main goal of our research was to provide a wide set of criteria based on a systematic literature review to assist the development of future MCDA tools in upper-middle-income countries for purchasing decisions of single-source innovative pharmaceuticals. Authors believe that the proposed criteria set can be a potential starting point for national adaptation in individual countries, which necessitates conclusion on how many criteria are applied at the national level and how the weight of each criterion is determined to reflect priorities of national health policies.

## Methods

### Sources of Information

A systematic literature review was conducted to identify value framework and MCDA articles listing criteria relevant for reimbursement level decision making of pharmaceuticals. The systematic literature review covered Scopus and Medline (via PubMed) databases. It was extended with a targeted literature review on specific databases of universities (Centre for Reviews and Dissemination (CRD) database; University of York, England; University of British Columbia, Canada) and websites of Health Technology Assessment Agencies (Independent Institute for Quality and Efficiency in Health Care (IQWIG), Germany; Agency for Health Quality and Assessment of Catalonia (AQUAS), Spain; Swedish Agency For Health Technology Assessment and Assessment Of Social Services (SBU), Sweden; National Institute for Health and Care Excellence (NICE), England; Scottish Medicines Consortium (SMC), Scotland; French National Authority for Health (HAS), France; Canadian Agency for Drugs and Technologies in Health (CADTH), Canada). Additionally, the following organizations’ websites were searched for eligible VFs and MCDAs: World Health Organisation (WHO) Health Evidence Network database; Institute for Clinical and Economic Review (ICER); European Union Projects (The Seventh Framework Programme (FP7), Horizon 2020 Programme, Innovative Medicines Initiative Joint Action Programme 2 (IMI2)); European Network for Health Technology Assessment (EUnetHTA); Health Technology Assessment International (HTAi); Innovation and Value Initiative (IVI); International Society for Pharmacoeconomics and Outcomes Research (ISPOR); European Patients’ Forum (EPF); European Federation of Pharmaceutical Industries and Associations (EFPIA); Pharmaceutical Research and Manufacturers of America (PhRMA); and the Society for Medical Decision Making. Registries of systematic literature reviews were also searched through PROSPERO and Cochrane Reviews databases.

### Search Limitations and Terms

The literature search was performed on the 28th of March 2019 for all databases. All articles on MCDA tools or VFs relevant for reimbursement level decision making on pharmaceuticals, listing a clear set of criteria and published since January 2013 were included. A restriction of publication date was used as the field of value frameworks has evolved remarkably in the past 5 years, and we wished to focus on more recent approaches, to capture relevant value elements in the present and near future. The search was also limited to English-language papers.

The first part of the search term was a combination of domains relevant to MCDA or VFs, in addition to the second term related to health care or pharmaceuticals. Synonyms and MeSH Terms of these two domains were used to conduct a comprehensive search in each database. The detailed search syntax is included in **Supplementary Figure 1**.

### Screening Phase

After deduplication, title and abstract screening and full-text screening were performed by two independent researchers. Disagreements between researchers were resolved by a principal researcher.

During the title and abstract screening phase, articles were excluded based on the following hierarchy: 1) Irrelevant title and no English abstract; 2) Not related to MCDA/VF OR MCDA/VF in other fields than human health care; 3) MCDA/VF in other fields than pharmaceuticals. Articles not fitting any of these exclusion criteria were eligible for full-text screening.

During full-text screening, articles were excluded if they fit in any of the following exclusion criteria: 1) Full text not found; 2) Abstract or commentary; 3) Duplicate; 4) Not English; 5) Non-pharmaceutical focus; 6) Not listing a clear set of criteria (e.g. purely methodological); 7) MCDA/VF supporting patient-level or shared decision making; 8) MCDA/VF supporting clinical guideline level/hospital level decision making; 9) Not original paper (e.g. framework already included in the review without major changes in criteria; literature review) or 10) Other specified reasons. Articles that did not fit any of the previous exclusion criteria were included in the data extraction phase. Other systematic literature reviews with a similar scope to this study were excluded by exclusion criterion 6, but were kept in a separate folder for snowball search.

### Data Extraction Phase

To systematically assess the findings, a standardized data extraction form was developed. The data extraction table and categorization principles for criteria were revised after a pilot data extraction phase. Qualitative research synthesis was used for presenting information, per the type of data. All criteria were extracted and clustered into seven predefined categories (disease-related; treatment-related; economic; societal; uniqueness and complexity; patient experience; other). All criteria related to the severity of disease, size of affected population, public health priorities or unmet need were considered disease-related. Treatment-related criteria were categorized as relating efficacy and/or safety of the treatment, or the strength of evidence of those. Criteria were considered as economic if they related to either the cost-effectiveness, budget impact, sustainability or potential for use outside of the reimbursed indication of the treatment. Additionally, organizational aspects of the assessed technology and the local investments of manufacturers were also considered as economic criteria. Societal criteria included equity aspects, prevention, productivity, caregiver burden, and legal aspects. Specific criteria for innovative technologies were categorized as uniqueness and complexity, including the innovative profile of the treatment and the complexity of manufacturing. Those criteria directly affecting patients’ experience other than those traditionally assessed in HTA (e.g. efficacy and safety) were categorized as patient experience. These criteria included patient convenience, adherence, and persistence, as well as value-added services.

The following information was extracted from each included study hence framework: 1) first author and year of publication; 2) country/region of (proposed) implementation; 3) organization developing the framework; 4) objective of the framework; 5) scope of use (e.g. general on all health technologies, oncologic drugs, orphan drugs); 6) name of criteria listed; 7) definition of each criterion, if available; 8) categorization of each criterion by the data extractor (into one of the 7 predefined categories); 9) scoring function of each criterion if available (which determines how a technology will be evaluated according to a particular criterion).

All extracted data were double-checked by a researcher other than the data extractor. Snowball search was performed to identify further relevant studies in included articles and in systematic literature reviews identified throughout the screening phases. When more than one article discussed the same framework, only the most recent article was included in the data extraction. Although, different adaptations of the same framework were included if criteria varied significantly.

### Quality Assessment

Identified frameworks were not assessed for quality, as the aim of the research was to collect potential value elements from recently published frameworks not to evaluate the current practice of the field.

### Merging and Selection Process of Criteria

When the data extraction phase was over, the merging and selection process of extracted criteria has started. First, criteria were deduplicated that of synonyms and substantially similar concepts. Then a multi-stakeholder team of experts went through all remaining criteria by the predefined categories in three rounds of workshops. Stakeholders involved were academics (2), HTA doers (2) and patient representatives (2) (**Table 1**).

**Table 1:** Detailed profiles stakeholders involved in the selection and merging process of criteria (n = 6).

**ACADEMIA**	A professor from an academic HTA Center with primer research focus on HTA methodologies
	An associate professor from an academic HTA Center with primer research focus on HTA methodologies
**HTA DOER**	A former HTA doer with 5 years of working experience at a national HTA body as a senior health economist
	An HTA doer, who coordinates large scale evidence synthesis projects
**PATIENT REPRESENTATIVE**	An oncology patient with thorough experience in validating patient reported outcome instruments
	A rare disease patient with a master’s degree in health economics and several years of experience in patient advocacy at national and international level

The merging and selection process was conducted in compliance with the ISPOR MCDA Emerging Good Practices Task Force report’s principles: completeness, non-redundancy, nonoverlaps, and preference independence (Marsh et al., 2016).

## Results

### Results of the Systematic Literature Review

**Figure 1:**
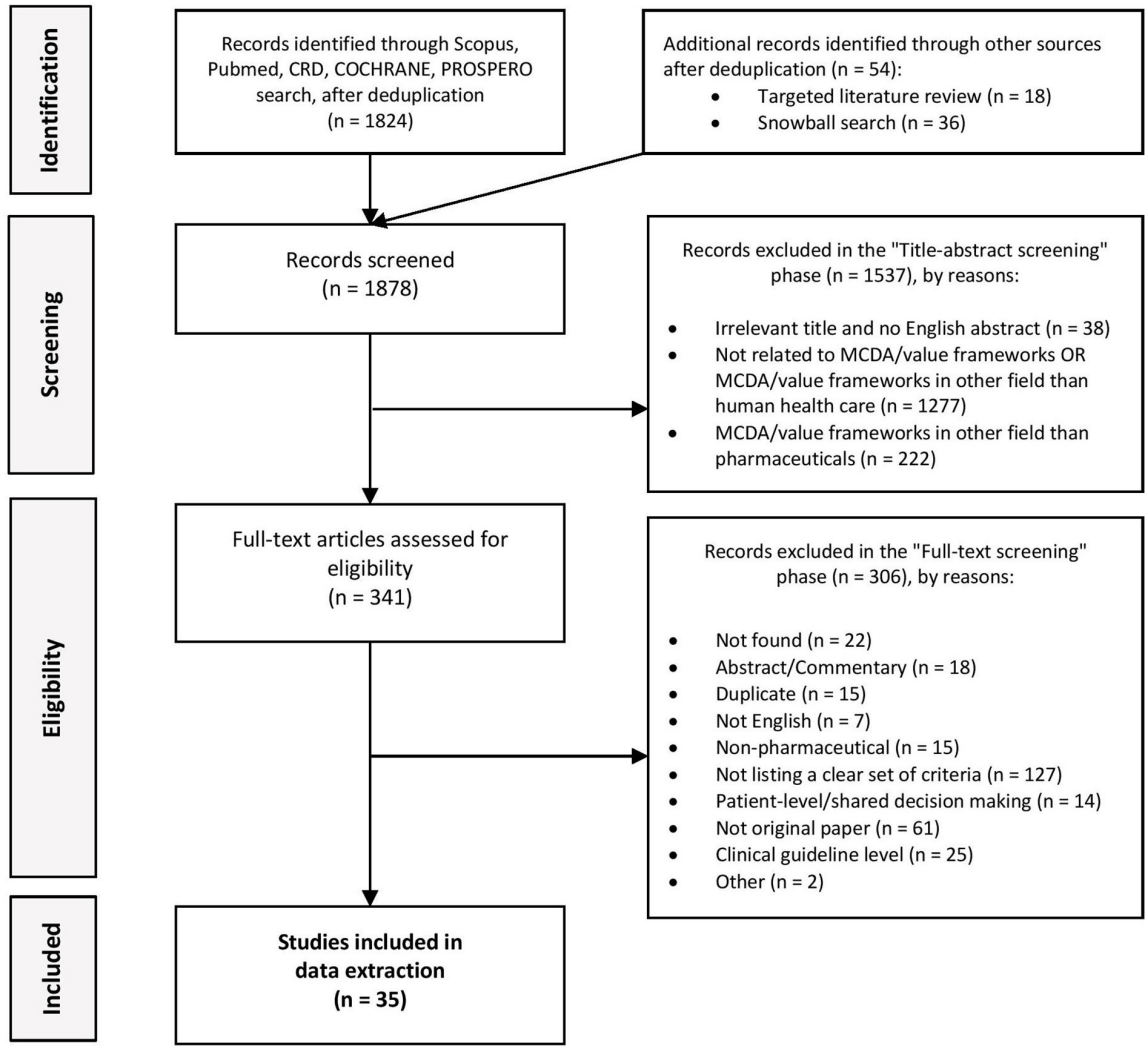
PRISMA flow diagram.

After the deduplication of results, a total of 1824 articles were identified through literature searches. The targeted literature search and the snowball method detected an additional 54 new records. Altogether, titles and abstracts of 1,878 records were screened from which 341 were eligible for the full-text screening phase. In total, 35 articles were considered to be eligible for the qualitative synthesis and were included in the data extraction phase (**Figure 1**).

A total of 37 different frameworks—MCDAs and VFs—were identified in the 35 articles. Based on the current World Bank Country and Lending rules for the 2020 fiscal year (World Bank Country and Lending Groups Country Classification, 2020), 9 were categorized as developed specifically for an upper-middle-income country. All others were meant to be implemented in a high-income country or were not specified other than continent or region (e.g. Europe, Latin-America, Central-Eastern Europe). Thirteen of the 37 had a general focus on all health technologies, 9 on medicines in general, while 6, 5 and 4 frameworks focused on orphan drugs, oncologic drugs, and off-patent drugs, respectively. The number of criteria listed ranged between 5 and 28, with an average of 11.3. About half of the frameworks (n=18) had explicit definitions of criteria listed, others published only the names of criteria. More than a third of the frameworks (n=15) had scoring functions, of which two had the same general scoring function proposed for all their criteria (e.g. 4-point scale (0–3)). About a third of the frameworks (n=14) had neither definitions nor scoring functions published for their criteria. Regarding the categories of criteria, all of them contained at least one treatment-related criterion. Economic and disease-related criteria were the second and third most prevalent, 86% (n = 32) and 81% (n = 30) of frameworks contained at least one of them, respectively. Fifty-nine percent (n=22) of value frameworks contained criteria categorized as societal, whereas 38% (n = 14) and 32% (n = 12) listed at least one criterion concerning the patient experience and the uniqueness and complexity of the treatment, respectively (**Table 2**).

**Table 2:** Summary table on the key results of the systematic literature review.

Author, Year	Country	Scope	Criteria								
			Number of criteria	Definitions	Scoring functions	Disease-related	Treatment-related	Economic	Societal	Uniqueness & complexity	Patient experience
Angelis and Kanavos, 2017	Europe	Medicines	28	yes	no	x	x	x	x	x	x
Annemans et al., 2017	Europe	Orphan drugs	17	yes	no	x	x	x	x		x
MSKCC, 2015	USA	Oncologic drugs	9	yes	yes	x	x	x		x	
Brixner et al., 2017	**Emerging countries**	Off-patent drugs	14	no	no		x	x			
CADTH, 2016	CAN	Oncologic drugs	8	yes	no	x	x	x			x
Cherny et al., 2015	Europe	Oncologic drugs	6	no	no		x				
Dankó and Molnár, 2017	Middle-income countries - CEE	General	5	yes	no		x	x	x		
Dionne et al., 2015	CAN	Medicines	10	yes	no	x	x	x	x	x	x
Doyle et al., 2019	USA	Oncologic drugs	5	no	no	x	x				
Drake et al., 2017	Latin-America	General	17	no	no	x	x	x	x		
MoCA, 2014	Europe	Orphan drugs	6	yes	yes	x	x				
Gilabert-Perramon et al., 2017	ESP - Catalonia	Orphan drugs	27	no	no	x	x	x	x		
Henshall and Schuller, 2013	Global	General	18	no	no	x	x	x	x	x	x
Howard et al., 2018	AUS	General	6	yes	yes	x	x	x	x	x	x
Hu et al., 2015	**CHN**	Off-patent drugs	10	no	no		x	x		x	x
ICER, 2017	USA	General	13	yes	yes*	x	x	x	x	x	
Inotai et al., 2018	IDN	Off-patent drugs	9	yes	yes		x	x	x		x
Iskrov et al., 2013	**BUL**	Medicines	18	no	yes	x	x	x	x		x
Iskrov et al., 2016	**BUL**	Orphan drugs	11	yes	yes	x	x	x	x		
Iskrov and Stefanov, 2016	**BUL**	Medicines	15	no	no	x	x	x	x		
Jaramillo et al., 2016	**COL**	General	15	yes	yes*	x	x	x	x		
Kolasa, 2014	POL	General	9	no	no	x	x	x	x		
Kolasa et al., 2016	POL	Orphan drugs	10	yes	yes	x	x	x		x	
Kristensen et al., 2017	Europe	General	9	no	no	x	x	x	x		x
Kwon et al., 2017	KOR	Oncologic drugs	8	yes	yes	x	x	x	x	x	
Lakdawalla et al., 2018	USA	General	12	yes	no	x	x	x	x	x	x
Oortwijn et al., 2017	Global	General	7	no	yes	x	x				
Radaelli et al., 2014	ITA - Lombardia	General	8	no	no	x	x	x	x		
Radu et al., 2016	**ROU**	Medicines	6	yes	yes		x	x			
Ramli et al., 2013	**MYS**	Medicines	12	no	no		x	x			x
Sussex et al., 2013	Europe	Orphan drugs	8	no	no	x	x		x	x	
Toumi and Rémuzat, 2017	Europe	Value added medicines	19	yes	no	x	x	x	x	x	x
Williams et al., 2014	UK	Medicines	10	no	yes	x	x	x			
Williams et al., 2014	ESP	Medicines	10	no	yes	x	x	x			
Williams et al., 2014	GER	Medicines	9	no	yes	x	x	x			
Youngkong, 2014	**THA**	General	9	yes	no	x	x	x	x		
Zah et al., 2015	Global	General	6	no	no	x	x	x			x

Abbreviations of upper-middle-income countries highlighted in bold.

*General, not criterion-specific scoring function.

AUS, Australia; AUT, Austria; BEL, Belgium; BUL, Bulgaria; CAN, Canada; CEE, Central and Eastern Europe; CHN, China; COL, Colombia; ESP, Spain; FRA, France; GER, Germany; GRE, Greece; IDN, Indonesia; ISR, Israel; ITA, Italy; KOR, Republic of Korea; MSKCC, Memorial Sloan Kettering Cancer Center; MYS, Malaysia; NED, the Netherlands; NOR, Norway; POL, Poland; POR, Portugal; ROU, Romania; SWE, Sweden; THA, Thailand; UK, United Kingdom; USA, United States of America.

### Results of the Merging and Selection Process of Criteria

**Figure 2:**
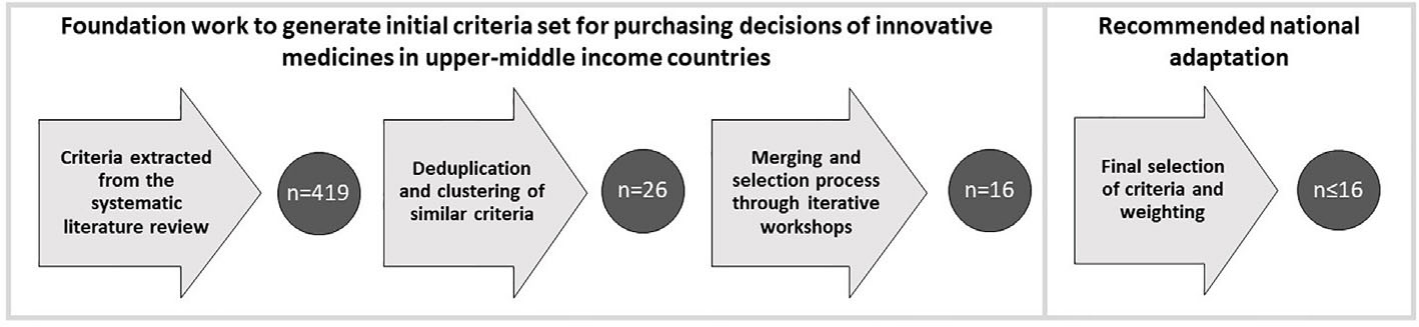
Flow of criteria through different phases of the foundation work and the recommended national adaption.

In total 419 criteria were extracted. After deduplication and clustering of similar criteria by each category, 26 criteria remained (**Figure 2**). These were the following: 1) disease-related criteria: severity of disease; size of affected population; unmet need; public health priority; 2) treatment-related criteria: efficacy; safety; strength of evidence; 3) economic criteria: cost; cost-effectiveness; budget impact; capacity; supply track record; potential use outside of the reimbursed indication(s); Local investment; 4) societal criteria: equity; prevention; productivity; caregiver burden; 5) uniqueness and complexity of treatment criteria: innovative profile of the treatment; manufacturing complexity; number of indications; spill-over effect; formulation; 6) patient experience criteria: patient convenience; patient adherence; value-added services.

Throughout the expert workshops, these 26 criteria were further merged to reduce overlaps and minimize the number of criteria proposed as possible. Criterions “efficacy” and “safety” were merged into a new criterion named “health gain.” The criterion called “size of affected population” was merged into criterion “equity,” thereby rare disease patients got listed as one aspect of the neglected populations. Meanwhile, the geographical access aspect of criterion “equity” (e.g. access for patients living in rural areas) was merged into criterion “availability and accessibility of alternative therapies.” Criterion “prevention” was merged into criterion “public health priority” as a potential focus of local public health policies, and it is highlighted as an example in the description of the new criterion. Likewise, the criterion concerning “caregiver burden” was merged into criterion “productivity,” where thereby the productivity loss of households, including both patients and caregivers, is taken into account. Also, the criterion “patient convenience” was merged into criterion “patient adherence and persistence” to reduce overlaps of capturing both effects and potential causes. Criterion “cost” was excluded because overlaps with criterion “cost-effectiveness” and criterion “capacity” was excluded because it is more health system-related than treatment-related. Criterion “number of indications,” “spill-over effect,” and “formulation” were excluded based on expert suggestions, to keep the initial set of criteria as curtail and condensed as possible. The expert workshops also explored potential incentives for the use of each criterion and proposed descriptions and examples of scoring functions. The final set of 16 criteria proposed for local adaptation is shown in **Table 3**.

**Table 3:** The proposed set of criteria with descriptions, potential incentives for inclusion and proposed scoring functions.

	Criterion	Incentive	Proposed scoring function
**DISEASE-RELATED**	**SEVERITY OF DISEASE** Prognosis and lifetime burden of illness	To reflect societies’ positive discrimination towards therapies for more severe diseases.	Chronic life-threatening (100%); acute life-threatening (80%); chronic with severe invalidity (60%); acute with severe invalidity (40%); other chronic diseases (20%); other acute diseases (0%)
	**AVAILABILITY AND ACCESSIBILITY OF ALTERNATIVE THERAPIES** Unmet medical need	To provide incentives for developing drugs targeting patients with real unmet need (i.e., e-health for non-city residents)	Patients have no current treatment (100%); patients have limited access to current treatment (50%); patients have access to effective and safe therapy (0%)
	**PUBLIC HEALTH PRIORITY** Local health system priorities, common goals, and specific interests	To incentivize therapies in diseases with high prevalence and significant public health burden (i.e., medical prevention)	Yes (100%); no (0%)
**TREATMENT-RELATED**	**HEALTH GAIN** Health benefits of using the pharmaceutical including improved quality of life, survival, clinical surrogate endpoints, and/or safety	To incentivize the development of drugs with an added benefit, either through better efficacy and/or better safety.	Therapeutic advancement compared to the standard of care (based on ASMR*): major (100%); important (75%); moderate (50%); minor (25%); no therapeutic advancement (0%)
	**STRENGTH OF EVIDENCE** Robustness of supporting clinical evidence, expert consensus, availability of evaluations from other countries	To incentivize the investment of manufacturers to improve the evidence base of new technologies both in clinical trials and in real world.	Evidence synthesis of RCTs plus real world data (100%); evidence synthesis of RCTs (80%); cohort studies (60%); case control studies (40%); case series/reports (20%); no evidence available (0%)
**ECONOMIC**	**COST-EFFECTIVENESS** Incremental cost-effectiveness ratio (ICER) compared to willingness to pay threshold	To incentivize the value for money aspect.	Dominant therapy (100%); below 3× GDP/capita (50%); Between 3× GDP/capita and 6× GDP/capita (50%–0% linear function); above 6x GDP/capita (0%); No data about cost-effectiveness (0%); **EXCLUSION CRITERIA**—the technology is dominated.
	**PROPORTION OF THE DRUG BUDGET** Total financial burden on the relevant health care budget(s)	To investigate the affordability of the medical technology.	How much is the proportion of the drug in the country’s annual drug budget? Saving money (100%); between 0% and 1% (100%–0% linear function); above 1% (0%)
	**POTENTIAL FOR USE OUTSIDE THE REIMBURSED INDICATION** Potential for off-label use of the drug, as a burden on the Payer	To disincentivize the utilization of the technology outside the reimbursed indication(s).	No potential for off-label used based on objective parameters or company guarantee (100%); Some potential for off-label use, patient selection is not based on an objective parameter (50%); Frequent off-label use predicted (0%)
	**SUPPLY TRACK RECORD** Sustainability of manufacturer business practices and supply track record assuring continuity	To incentivize supply reliability.	Manufacturer is financially capable and willing to guarantee supply (100%); No precedence of supply problems in the last 5 years (80%); Single precedence of supply problems in the last 5 years (50%); minor and fairly frequent problems in the last 5 years (20%); major and multiple problems in the last 5 years (0%)
	**LOCAL INVESTMENT** Potential for the manufacturer to make local investment, supporting the local economy	To incentivize manufacturers to invest into the local economy.	The manufacturer has significant local investment in the country (100%); The manufacturer has moderate local investment in the country (67%); The manufacturer has minor local investment in the country (33%); The manufacturer has no local investment in the country (0%)
**SOCIETAL**	**EQUITY** Reducing health disparities	To incentivize medical therapies to neglected populations (pediatric patients, rare diseases, pregnancy).	Ultra-orphan disease (100%); indication for paediatric, rare, pregnant, or psychiatric patients (75%); none of the above (0%)
	**PRODUCTIVITY** Financial protection of households through improved productivity, reduced caregiver burden, or avoided patient cost (reduction in the medical costs of the patients)	To incentivize therapies improving the productivity of patients and/or caregivers.	Improvement in the financial status of the household: Major (100%); minor (50%); no improvement (0%); no data (0%)
**UNIQUENESS & COMPLEXITY**	**INNOVATIVE PROFILE OF TREATMENT** First in class therapies	To incentivize companies to develop novel technologies instead of me-too products.	Is it a first in class therapy? Yes (100%); no (0%)
	**MANUFACTURING COMPLEXITY** The complexity of the manufacturing technology	To appreciate efforts of manufacturers to develop innovative products requiring complex manufacturing processes.	Manufacturing technology complexity: expensive biotechnological processes (100%); complex synthetic path consisting of at least three independent chemical transformation; (50%); manufacturing requires the use of separation techniques for most intermediates (50%); anything else (0%)
**PATIENT EXPERIENCE**	**PATIENT ADHERENCE AND PERSISTENCE** Potential improvements in adherence and persistence of patients taking the pharmaceutical	To incentivize potential improvements in the adherence and/or persistence of patients using the medical technology due to improved patient convenience, tolerability, etc.	Treatment improves adherence and/or persistence: Significantly (100%); moderately (50%); no potential improvement (0%)
	**VALUE ADDED SERVICES TO PATIENTS** Additional services provided by the manufacturer to patients	To incentivize efforts of manufacturers to improve patient experience through transparently provided added value services (i.e., mobile application, disease awareness, patient education)	Company provides multiple value-added services, e.g., disease awareness and education (100%); company provides one single value-added service (50%); company does not provide value-added services (0%)

*ASMR is a five-level scale used by the French National Authority for Health (HAS) used to evaluate the incremental or added value of the product.

## Discussion

To the best of our knowledge, this is the first publication supporting the creation of MCDA for the reimbursement of innovative pharmaceuticals in upper-middle-income countries. Similar publications identified throughout our systematic literature review were a systematic review on the use of methodological frameworks to set health care priorities in low-income and lower middle-income countries (Wiseman et al., 2016), MCDA tools specific to one certain upper-middle-income country with either a specific or a general scope (Iskrov et al., 2013; Ramli et al., 2013; Youngkong, 2014; Hu et al., 2015; Iskrov and Stefanov, 2016; Jaramillo et al., 2016; Radu et al., 2016) and an MCDA tool for emerging countries focusing on the evaluation of off-patent pharmaceuticals (Brixner et al., 2017).

The proposed set of 16 criteria provides a comprehensive initial framework that can be adapted to upper-middle-income countries’ circumstances based on local priorities. As the literature review indicated, there is increasing attention to criteria concerning patient experience. To emphasize its significance, two independent criteria are proposed to capture this emerging aspect. Criteria concerning the uniqueness and complexity of the therapy were included as important aspects of innovative medicines, especially in the light of recently launched genetic therapies.

The merging and selection process aimed to minimize the number and magnitude of overlaps in the framework to prevent double-counting. However, some overlap is inevitable in any MCDA. For example, a potential remaining overlap might be between criteria “patient adherence and persistence” and “value added services”. Even though value-added services can potentially improve patient adherence and persistence, the importance of these services should be emphasized.

The core of an MCDA decision-supporting tool for repeated use is in its availability of increasing the transparency of decision making. When MCDAs are published, but without the detailed definitions and scoring functions of the criteria they may fail to fulfill this purpose. With almost half of the identified frameworks not publishing either definitions or scoring functions, we thought it was an absolute must to propose one for each criterion, even if they can be further modified during local adoption. This approach hopefully further supports the interpretation thereby the implementation of our work.

### Previous Systematic Literature Reviews With a Similar Scope

Throughout the literature review, nine previous systematic literature reviews of MCDAs and VFs were identified. The most recent by Kolasa et al. present the then-current state of knowledge concerning the MCDA utilization in the value assessment of drug therapies. They identified 18 MCDAs in April 2017 concerning pricing and reimbursement level decision making of pharmaceuticals, similarly to the present review. Kolasa and colleagues revealed several shortcomings in the implementation of MCDA to health care decision–making, like the lack of consistency of approaches chosen toward the type and the number of stakeholders involved in the MCDA experiments. Similarly to our findings, they also noted that none of the reviewed studies have reported how MCDA results impacted the real-life settings, and that examples of MCDA’s successful launch in a real-life decision-making process are genuinely scarce (Kolasa et al., 2018). Another recent systematic review by Frazão and colleagues aimed to analyze and synthesize articles found in the literature on applying MCDA to health care while assessing general issues and methodological aspects. They analyzed the trends in MCDA publications in addition to the types of sources and methods used to develop particular parts of the MCDAs. Based on their review, they identified a growing trend in the application of MCDA in the field of health, with great emphasis from the year 2014 (Frazão et al., 2018). This finding is in line with our approach of restricting the timeframe of our research to 2013 and beyond. Another systematic review aimed to summarize the evaluation criteria of orphan medicines with a regional scope, particularly for Central and Eastern European countries (Zelei et al., 2016). The authors concluded that due to external price referencing of pharmaceuticals, the relative budget impact of orphan drugs is expected to be higher in CEE than in Western European (WE) countries unless accessibility of patients remains more limited in poorer European regions. This observation may apply for other innovative medicines and upper middle-income countries too, further strengthening the need for locally adopted MCDA tools.

### Recommendations for Adapting the Study Results

The maximum number of criteria in an MCDA is recommended to set around 10, otherwise too many criteria might mean too little weight for each criterion (Inotai et al., 2018b). Hence, we suggest selecting approximately 10 of our 16 proposed criteria during the adaptation period, based on local priorities (**Figure 2**). Health care system structure, decision-making framework, the health status of the population, financial backgrounds, and several other key factors can differ significantly across countries, making transferability a key issue in health care decision-making (Goeree et al., 2011). Just like reimbursement recommendations of HTA bodies cannot be used for decision making in other countries without considering transferability (Kaló et al., 2012), the same applies for constructing MCDAs. It has been strongly recommended that MCDA frameworks should only contain locally relevant criteria, taking into close consideration local feasibility, as local experts are always more familiar with specific problems, health care priorities and processes of the particular country than international experts. Furthermore, the development of a new MCDA should be based on the decision-making criteria that are currently used in particular countries (Inotai et al., 2018b). Some steps of MCDA development identified by the second ISPOR Task Force report (Marsh et al., 2016) can be conducted by a small number of experts and policymakers (e.g. defining the decision problem), while others (e.g., selecting and structuring criteria) necessitate a wider, multi-stakeholder approach. Incorporating the preferences of various stakeholders can be conducted at specifically designed workshops (Inotai et al., 2018b), though the results of previously conducted desk research can be a good starting point in all cases. Our research aims to provide a background for such future implementations.

### Potential Limitations of MCDA Use

Like all methodologies, MCDA has potential limitations. A scoping review of different priority setting approaches found that while the intention of developing such tools is for them to eventually be used to guide routine policymaking, not many have been integrated into routine practice. Cited limitations included the technical complexity of the approaches and resource demand (Kapiriri and Razavi, 2017). The lack of and need for publishing the real-word experience of using MCDAs has been highlighted by other researchers (Kapiriri and Razavi, 2017; Kolasa et al., 2018) and it is in line with our concerns as well. Such publicly available information can be crucial in supporting the implementation of MCDA in more countries and should be highly encouraged to publish. Another example of potential limitations of MCDA that has been discussed arises when incorporating cost-based criteria. Critics believe that the preferences of technology providers or users may not be the appropriate basis for evaluating the efficiency of technologies as the benefit that may be forgone is more prevalent in their thinking than the willingness to pay (WTP) of third-party payers. And even if the exercise aims to find the WTP with broad and imprecise definitions of criteria, its appropriateness can be questionable (Marsh et al., 2018). Another critique of the stakeholder involvement in the development of MCDAs is that when reviewed, there were no common patterns in the types and the size of the study population of involved stakeholders (Kolasa et al., 2018). Key issues of real-world feasibility may also emerge from the inappropriate selection of an analysis method, wrong interpretation of results, and using software solutions that are not widely available, as the results of the criteria ranking can depend on the various MCDA models that are being used (Kujawski, 2003). This is part of the reason why MCDA and its development method should be simple and easy to understand (Inotai et al., 2018b), and the trade-off between scientific accuracy and resource intensity and general participant burden should be taken into account in case of choosing between criteria weighting methods (Németh et al., 2019). To interpret the results of an MCDA evaluation easier, Angelis and Kanavos suggested a framework in which the aggregate value score (that does not include cost-related criteria) produced by the MCDA process would be the benefit component. The so-called incremental cost value ratio (ICVR), the purchasing cost per incremental MCDA value unit gained could be used similarly as the incremental cost-effectiveness ratio (ICER) is applied in several countries (Angelis and Kanavos, 2016).

### Limitations of the Study

Limitations of the present research include, that only published sets of criteria were taken into account. Therefore, the criteria being used in confidential decision-making frameworks could not be incorporated into the analysis. In addition, the categorization of criteria was conducted based on personal judgments of researchers. We tried to minimize the personal variability by 1) creating an objective categorization guideline during the pilot data extraction phase and 2) by categorizing all criteria by two independent researchers and resolving conflicts by a principal researcher. A general limitation was, that a significant amount of frameworks (n=14) had nether descriptions, nor scoring functions published for their criteria listed as value elements. Therefore, the precise interpretation of certain criteria gathered from the literature was challenging and forms a source of uncertainty. Finally, as MCDA frameworks should always be adapted to the local environment and the particular decision problem, it is possible that some special cases may require additional specific criteria. Therefore, our set of 16 criteria should be used as a starting point only.

Future research can focus on MCDAs developed for other fields of health care and other decision problems. Since the theoretical side of MCDA has been relatively well-researched during the last few decades, more emphasis could be put on the practical side of MCDA implementation, based on real-life examples.

## Conclusion

Based on the results of the systematic literature review, we established a pool of 16 criteria that can serve as a basis for constructing MCDAs to inform reimbursement decision making of innovative pharmaceuticals, especially in upper middle-income countries. The feasibility of implementation and adaptation to the local context are key aspects that should be taken into account in all cases. When adapting to the circumstances of a certain upper-middle-income country, we suggest setting up a group of local stakeholders to select and weigh not more than 10 of the 16 criteria based on local priorities. Well-defined descriptions and scoring functions of the criteria are inevitable for objective and transparent decision making. Further research is needed on the real-life examples of MCDAs as literature on the experiences after MCDA implementation is scarce and incomplete.

## Author Contributions

Conception and design: ZK, BN, IJ, HT, JS. Analysis and interpretation of data: ZK, BN, IJ, BE, HT, MK. Drafting of the manuscript: IJ, BN, BE. Critical revision of the manuscript for important intellectual content: ZK, MK, HK, SA. Supervision: JS, ZK, SA.

## Funding

The study was funded by Novartis International AG.

## Conflict of Interest

The authors declare that the research was conducted in the absence of any commercial or financial relationships that could be construed as a potential conflict of interest.

This work was supported in part by a contract with Novartis International AG. The funding agreement ensured the authors’ independence in designing the study, interpreting the data, writing, and publishing the report. The content of the paper and the conclusions are those of each author and cannot be understood as reflecting those of the organization that employs them.
