# Supplementary material for: Potential Criteria for Frameworks to Support the Evaluation of Innovative Medicines in Upper Middle-Income Countries—A Systematic Literature Review on Value Frameworks and Multi-Criteria Decision Analyses
Source: Front Pharmacol. 2020 Aug 14;11:1203. doi: 10.3389/fphar.2020.01203 (PMC7456841; doi:10.3389/fphar.2020.01203)
Supplement: Supplementary file 1 [file DataSheet_1.docx]

Supplementary Material

**Supplementary Figure 1 – Search strategy**

("multicriteria decision analysis"[Title/Abstract] OR MCDA[Title/Abstract] OR "multiple criteria decision aiding"[Title/Abstract] OR "multicriteria decision making"[Title/Abstract] OR "MCDM"[Title/Abstract] OR "multicriteria analysis"[Title/Abstract] OR "multi-attribute decision analysis"[Title/Abstract] OR "value framework"[Title/Abstract] OR] OR "value assessment"[Title/Abstract])

AND

("health"[Title/Abstract] OR "health care"[Title/Abstract] OR "healthcare"[Title/Abstract] OR "medical decision"[Title/Abstract] OR "medical decision making"[Title/Abstract] OR "medicine"[Title/Abstract] OR "medication"[Title/Abstract] OR "disease"[Title/Abstract] OR "pharmacy"[Title/Abstract] OR "pharmaceutical"[Title/Abstract] OR drug[Title/Abstract] OR therapy[Title/Abstract] OR therapies[Title/Abstract] OR treatment[Title/Abstract] OR “medical technology” [Title/Abstract])

AND

English[Language])

AND

("2013/01/01"[Date - Publication] : "2019/03/28"[Date - Publication])

**
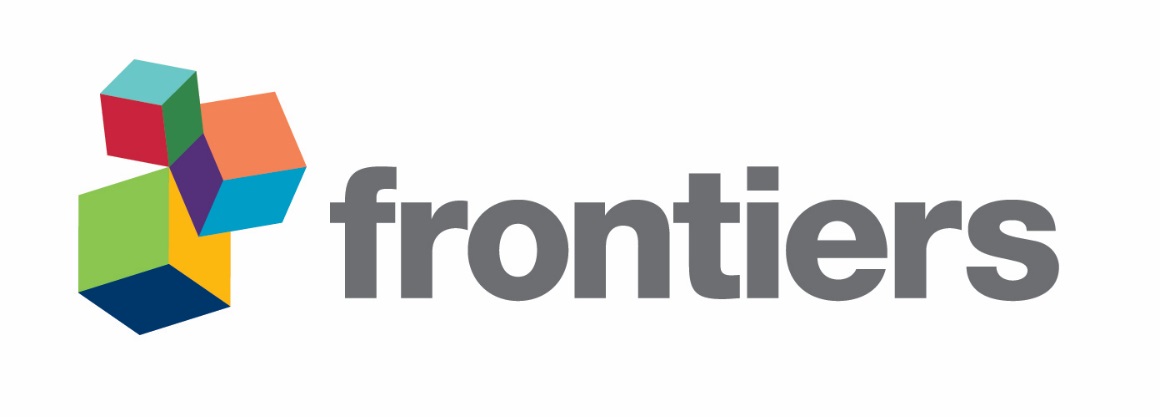
**
